# Bayesian adaptive randomized trial of blended cognitive behavioral therapy for severe fatigue in stable diffuse glioma

**DOI:** 10.1093/neuonc/noaf256

**Published:** 2025-11-09

**Authors:** Maxine Gorter, Jantine G Röttgering, Vera Belgers, Marieke E C Blom, Brigit Thomassen, Philip C De Witt Hamer, Johanna M Niers, Mathilde C M Kouwenhoven, Henri P Bienfait, Celine S Gathier, Annette Compter, Marjolein Geurts, Tom J Snijders, Peter M van de Ven, Linda Douw, Hans Knoop, Martin Klein

**Affiliations:** Amsterdam UMC location Vrije Universiteit Amsterdam, Anatomy and Neurosciences, De Boelelaan 1117, Amsterdam, The Netherlands (M.G.O., M.E.C.B., L.D.); Cancer Center Amsterdam, Brain Tumor Center, Amsterdam, The Netherlands (M.G.O., J.G.R., V.B., M.E.C.B., B.T., P.C.D.W.H., J.M.N., M.C.M.K., L.D., M.K.); Cancer Center Amsterdam, Brain Tumor Center, Amsterdam, The Netherlands (M.G.O., J.G.R., V.B., M.E.C.B., B.T., P.C.D.W.H., J.M.N., M.C.M.K., L.D., M.K.); Amsterdam UMC location Vrije Universiteit Amsterdam, Medical Psychology, De Boelelaan 1117, Amsterdam, The Netherlands (J.G.R., M.K.); Cancer Center Amsterdam, Brain Tumor Center, Amsterdam, The Netherlands (M.G.O., J.G.R., V.B., M.E.C.B., B.T., P.C.D.W.H., J.M.N., M.C.M.K., L.D., M.K.); Amsterdam UMC location Vrije Universiteit Amsterdam, Neurology, De Boelelaan 1117, Amsterdam, The Netherlands (V.B., B.T., J.M.N., M.C.M.K.); Amsterdam UMC location Vrije Universiteit Amsterdam, Anatomy and Neurosciences, De Boelelaan 1117, Amsterdam, The Netherlands (M.G.O., M.E.C.B., L.D.); Cancer Center Amsterdam, Brain Tumor Center, Amsterdam, The Netherlands (M.G.O., J.G.R., V.B., M.E.C.B., B.T., P.C.D.W.H., J.M.N., M.C.M.K., L.D., M.K.); Cancer Center Amsterdam, Brain Tumor Center, Amsterdam, The Netherlands (M.G.O., J.G.R., V.B., M.E.C.B., B.T., P.C.D.W.H., J.M.N., M.C.M.K., L.D., M.K.); Amsterdam UMC location Vrije Universiteit Amsterdam, Neurology, De Boelelaan 1117, Amsterdam, The Netherlands (V.B., B.T., J.M.N., M.C.M.K.); Cancer Center Amsterdam, Brain Tumor Center, Amsterdam, The Netherlands (M.G.O., J.G.R., V.B., M.E.C.B., B.T., P.C.D.W.H., J.M.N., M.C.M.K., L.D., M.K.); Amsterdam UMC location Vrije Universiteit Amsterdam, Neurosurgery, De Boelelaan 1117, Amsterdam, The Netherlands (P.C.D.W.H.); Cancer Center Amsterdam, Brain Tumor Center, Amsterdam, The Netherlands (M.G.O., J.G.R., V.B., M.E.C.B., B.T., P.C.D.W.H., J.M.N., M.C.M.K., L.D., M.K.); Amsterdam UMC location Vrije Universiteit Amsterdam, Neurology, De Boelelaan 1117, Amsterdam, The Netherlands (V.B., B.T., J.M.N., M.C.M.K.); Cancer Center Amsterdam, Brain Tumor Center, Amsterdam, The Netherlands (M.G.O., J.G.R., V.B., M.E.C.B., B.T., P.C.D.W.H., J.M.N., M.C.M.K., L.D., M.K.); Amsterdam UMC location Vrije Universiteit Amsterdam, Neurology, De Boelelaan 1117, Amsterdam, The Netherlands (V.B., B.T., J.M.N., M.C.M.K.); Gelre Ziekenhuizen Apeldoorn, Neurology, Apeldoorn, The Netherlands (H.P.B.); Elisabeth-TweeSteden Hospital, Neurology, Tilburg, The Netherlands (C.S.G.); Netherlands Cancer Institute—Antoni van Leeuwenhoek, Department of Neurology, Amsterdam, The Netherlands (A.C.); Department of Neurology, Brain Tumor Center, Erasmus MC Cancer Institute, Rotterdam, The Netherlands (M.G.); Department of Medical Oncology, Erasmus MC Cancer Institute, Rotterdam, The Netherlands (M.G.); UMC Utrecht Brain Center, University Medical Center Utrecht, Neurology and Neurosurgery, Utrecht, The Netherlands (T.J.S.); Julius Center for Health Sciences and Primary Care, University Medical Center Utrecht, Data and Biostatistics, Utrecht, The Netherlands (P.M.v.d.V.); Amsterdam UMC location Vrije Universiteit Amsterdam, Anatomy and Neurosciences, De Boelelaan 1117, Amsterdam, The Netherlands (M.G.O., M.E.C.B., L.D.); Cancer Center Amsterdam, Brain Tumor Center, Amsterdam, The Netherlands (M.G.O., J.G.R., V.B., M.E.C.B., B.T., P.C.D.W.H., J.M.N., M.C.M.K., L.D., M.K.); Amsterdam UMC location University of Amsterdam, Medical Psychology, Meibergdreef 9, Amsterdam, The Netherlands (H.K.); Amsterdam Public Health Research Institute, Expert Center for Chronic Fatigue, Amsterdam, The Netherlands (H.K.); Division of Psychology, Department of Clinical Neuroscience, Karolinska Institutet, Stockholm, Sweden (H.K.); Cancer Center Amsterdam, Brain Tumor Center, Amsterdam, The Netherlands (M.G.O., J.G.R., V.B., M.E.C.B., B.T., P.C.D.W.H., J.M.N., M.C.M.K., L.D., M.K.); Amsterdam UMC location Vrije Universiteit Amsterdam, Medical Psychology, De Boelelaan 1117, Amsterdam, The Netherlands (J.G.R., M.K.)

**Keywords:** blended cognitive behavioral therapy, diffuse glioma, fatigue, quality of life

## Abstract

**Background:**

While severe fatigue is common in patients with diffuse glioma, no evidence-based treatment is currently available. The objective of this RCT was to evaluate the efficacy of blended cognitive behavioral therapy (bCBT) for severe fatigue.

**Methods:**

Severely fatigued patients (Checklist Individual Strength, fatigue-severity subscale [CIS-fatigue] ≥ 35) with diffuse glioma and stable disease were randomized to 12 weeks of bCBT or a waiting list condition (WLC). The primary endpoint was fatigue severity 2 weeks after intervention. This Bayesian adaptive trial included prespecified interim analyses for efficacy at *n* = 40, 50, 60, 70, and 80. Secondary outcomes—health-related quality of life (HRQoL), anxiety, future uncertainty, and depression—were assessed at 2 and 12 weeks after intervention.

**Results:**

The trial was stopped for efficacy at the first interim analysis. Of 47 patients randomized, 40 patients reached the primary endpoint (mean age 53 years, 47% female). The posterior probability that CIS-fatigue scores were lower with bCBT than with WLC was 99.94%, with a large standardized effect size (Cohen’s *d*) of 1.12 [95% CI: 0.43–1.81]. At 2 weeks after intervention, 68% of patients were no longer severely fatigued after bCBT, compared to 24% in WLC. At 12 weeks follow-up, fatigue was still significantly lower in the bCBT group compared to WLC (*d *= 1.22). bCBT also demonstrated beneficial effects (*d *= 0.42-1.19) on anxiety, HRQoL, and future uncertainty.

**Conclusions:**

bCBT significantly reduces fatigue and improves anxiety and HRQoL in patients with diffuse glioma. These findings enable evidence-based supportive care strategies for reducing fatigue and enhancing HRQoL in this population.

Key Points

- Blended cognitive behavioral therapy (bCBT) is effective in reducing severe fatigue in diffuse glioma.
- bCBT also improves anxiety, future uncertainty, and health-related quality of life.
- Positive effects of bCBT sustained for 12 weeks after the intervention.

Importance of the Study

This study demonstrates the effect of blended cognitive behavioral therapy (bCBT) to reduce severe fatigue among patients with diffuse glioma. Compared to a waiting list control condition, bCBT also reduces anxiety, future uncertainty, and improves health-related quality of life, establishing the evidence-base for this treatment in neuro-oncological care.

Despite intensive treatment with surgery, chemotherapy, and radiotherapy, diffuse gliomas exhibit continuous growth, ultimately leading to death. Throughout the disease course, patients frequently experience a range of symptoms, including neurological deficits, cognitive impairment, depression, and fatigue that constitute a substantial symptom burden and a reduced health-related quality of life (HRQoL).^1-3^ Fatigue is one of the most frequently reported symptoms, and patients describe their fatigue as the most burdensome of all symptoms.^4^ Up to 96% of patients with diffuse glioma report severe fatigue at various stages of the disease.^5-10^ While demographic, biomedical, neuropsychological, psychosocial, and behavioral factors contribute to the development and persistence of fatigue,^11^ its etiology in brain tumor patients is still poorly understood. Higher levels of fatigue are linked to reduced self-perceived cognitive functioning and an increase in psychological symptoms including depression, anxiety, and sleep disturbances, known to significantly impair daily role functioning and overall HRQoL.^6^^,^^8^^,^^11^^,^^12^

Currently, there are no evidence-based pharmacological or behavioral interventions available that specifically target fatigue in brain tumor patients, despite the expectation that effective treatment may improve HRQoL.^1^^,^^11^ A 2022 Cochrane review identified only 3 randomized controlled trials (RCTs) that exclusively enrolled severely fatigued patients and designated fatigue as the primary outcome.^7^ All 3 trials investigated pharmacologic agents, namely modafinil, dexamphetamine sulfate, and armodafinil, but none demonstrated efficacy over a placebo.^13-15^ Behavioral interventions have been insufficiently studied in this population. A study into the feasibility of recruitment and retention of RCTs, consisting of 2 novel lifestyle coaching programs in severely fatigued glioma, reported preliminary benefits on fatigue and mental health outcomes.^16^ Beyond these limited efforts, several studies in heterogeneous brain tumor populations have examined a range of interventions, including pharmacologic agents,^17-22^ educational programs,^23^ cognitive rehabilitation,^24^^,^^25^ exercise-based therapies,^26-30^ and internet-guided self-help.^31^ Results have been mixed: while some studies reported improvements in fatigue,^20-22^^,^^24^^,^^26^^,^^27^^,^^31^ others found no benefit,^17-19^^,^^23^^,^^25^^,^^28^^,^^30^ or even worsening of symptoms.^29^ Importantly, these studies did not restrict inclusion to severely fatigued diffuse glioma patients nor prioritize fatigue as the primary endpoint.

Cognitive behavioral therapy (CBT) has proven effective for treating persistent fatigue in non-central nervous system (CNS) cancer survivors and patients in the palliative phase.^32^^,^^33^ Fatigue-specific CBT targets perpetuating factors like sleep disturbances and maladaptive thoughts, which sustain fatigue beyond the initial impact of cancer and its treatment.^34^^,^^35^ In a RCT by our group, a 12-week CBT program significantly reduced fatigue in severely fatigued palliative patients with breast, colorectal, or prostate cancer.^36^ Additionally, CBT led to improvements in both fatigue and self-perceived cognitive dysfunction among survivors of breast, testicular, other solid tumors, and hematologic cancers.^37^ Outside the oncology field, CBT has also proven effective in managing fatigue in conditions such as multiple sclerosis,^38^ stroke,^39^ and traumatic brain injury.^40^

Blended CBT (bCBT), combining online modules with face-to-face sessions enhances accessibility, flexibility, and engagement while improving treatment efficiency, reducing costs, and maintaining continuity of care.^41^ While efficacy of bCBT has been demonstrated in breast cancer survivors,^42^^,^^43^ it is unknown whether fatigued patients with diffuse glioma also benefit from bCBT. In this context, a recent trial of telehealth group CBT for insomnia in primary brain tumor patients demonstrated not only improved sleep but also reduced fatigue,^44^ supporting the potential value of bCBT-based approaches in this population. Therefore, this RCT tested whether a 12-week bCBT program reduces fatigue compared with a waiting list control in patients with diffuse glioma who report severe fatigue. Secondary objectives were to assess effects on HRQoL, anxiety, future uncertainty, and depression at 2 and 12 weeks post-intervention.

## Methods

### Study Design

We conducted a randomized controlled trial recruiting adults with severe fatigue and stable diffuse glioma between January 2, 2020 and May 2, 2024. The published trial protocol^45^ was approved by the ethical review board of the Amsterdam University Medical Center (METc 2019.714). All participants provided written informed consent prior to study participation.

### Patient Population

Patients were included if they were in a stable disease phase. This period is typically characterized by the absence of tumor progression and anti-tumor treatment, and therefore a reduced frequency of hospital visits, providing a window to focus on quality-of-life interventions. Eligible patients had histologically confirmed diffuse glioma WHO CNS5 (2021) grade 2-4, irrespective of IDH status, and were clinically and radiologically stable (ie, no oncological treatment for at least 8 weeks and no signs of tumor progression at time of inclusion). Patients were ≥18 years old, reported severe fatigue (Checklist Individual Strength, fatigue severity subscale [CIS-fatigue] score ≥ 35),^46^ had no other underlying somatic cause for fatigue other than diffuse glioma or its treatment after blood screening for other causes, and were expected to survive for 12 weeks or longer. We excluded patients with suspected depressive disorder, primary sleep disorders, poor performance status (Karnofsky performance scale score <70), any corticosteroid use, or those currently receiving (pharmacological) treatment for fatigue or any mental disorder. Suspected depressive disorder was screened with the Beck Depression Inventory for primary care (BDI-pc),^47^ if scores exceeded 4, further assessed using the Mini-International Neuropsychiatric Interview (MINI) depression module.^48^ Primary sleep disorders were assessed by patient self-report of a formal diagnosis. A complete overview of the eligibility criteria can be found in the published protocol.^45^

### Recruitment, Randomization, Procedures, Blinding, and Intervention

Patients were consecutively identified in routine clinical practice by healthcare professionals involved in brain tumor care (neurologists, neurosurgeons, radiation oncologists, nurse practitioners, oncologists, and psychologists) at the Brain Tumor Center Amsterdam and collaborating Dutch hospitals. After providing written informed consent and completing the eligibility check, patients completed baseline measurements, including questionnaires, and were randomized to blended cognitive behavioral therapy (bCBT) or to a waiting list control group (WLC). Randomization was performed via a secure web-based program using block randomization with variable block sizes of 2 and 4, ensuring balanced allocation between the intervention and control groups. All patients received standard care in accordance with national glioma clinical practice guidelines. Patients were instructed not to follow any other interventions targeting fatigue. Although blinding of patients was not possible, outcome assessors of study outcomes were blinded to treatment allocation.

#### Blended Cognitive Behavioral Therapy

A detailed description of the intervention is provided in the published protocol^45^ and a concise summary of the intervention modules is provided in the Supplementary Material. In short, bCBT is an intervention consisting of therapist-led sessions and online modules with therapist feedback. The 12-week program consisted of 5 patient–therapist sessions, delivered either face-to-face or online via secure video consultation, depending on patient preference. bCBT for fatigue is a structured, goal-oriented therapy that targets fatigue-perpetuating factors such as unhelpful thoughts, disrupted sleep, and unbalanced activity levels. It combines psychoeducation, behavioral activation and graded activity, cognitive restructuring, and self-monitoring to help patients better understand, manage, and reduce their fatigue.^45^ Treatment was provided by trained and experienced cognitive-behavioral therapists, at a tertiary treatment center for Chronic Fatigue, Amsterdam UMC.

##### Waiting List Control Group

Patients assigned to the WLC group were on a waiting list and were offered the opportunity to do the bCBT program after the study period (ie, after completing questionnaires of the follow-up measurement, 12 weeks after intervention).

### Outcome Measures

#### Primary Outcome

The primary outcome measure was the fatigue severity subscale score (CIS-fatigue) of the Checklist Individual Strength (CIS-20)^46^ 2 weeks post-intervention. The CIS-20 is a 20-item self-report questionnaire measuring fatigue severity and fatigue-related aspects such as difficulty concentrating or problem with staying physically active. Each item is rated on a 7-point Likert scale, with higher scores indicating more severe problems. The CIS-fatigue subscale (8 items; range 8-56) classifies severe fatigue at ≥35.^46^

#### Secondary Outcomes

Secondary outcomes were assessed at baseline, post-intervention (2 weeks after intervention), and follow-up (12 weeks after intervention) and included: fatigue assessed with the Fatigue Severity Scale (FSS; higher scores = more fatigue)^49^; HRQoL with European Organization for Research and Treatment of Cancer-Quality of Life Questionnaire Core 30 Global Health Status/Quality of Life scale (EORTC-QLQ-C30 Global Health Status/QoL scale; higher scores = better HRQoL)^50^; Anxiety with Beck Anxiety Inventory (BAI)^51^ and depression with the BDI-pc^47^ (both higher scores = more symptoms). Lastly, Future Uncertainty was assessed with the EORTC Brain Neoplasm module Future Uncertainty scale (EORTC QLQ-BN20 FU).^52^ This scale consists of items 31-33, 35 (feeling uncertain about the future, fear of physical decline, worry about disruption of family life, and a more pessimistic outlook on the future) and were scored from 0 to 100, with higher scores indicating greater uncertainty. Patients who discontinued the study or intervention were asked to complete post-intervention and follow-up assessments per intention-to-treat approach.

Acceptance and perceived burden of the intervention were assessed with a brief evaluation questionnaire completed directly after the intervention and at follow-up. Patients rated their overall satisfaction with the intervention on a 0-10 scale (0 = very dissatisfied, 10 = very satisfied) and were invited to provide open comments regarding their experiences (eg, perceived helpful elements, suggestions for improvement, or reasons for discontinuation). Also, patients who discontinued the intervention were invited to provide qualitative feedback.

### Sample Size

The study used a Bayesian multistage trial design with equal randomization and repeated interim evaluations for efficacy and futility. Interim analyses were planned when outcomes were available for 40, 50, 60, 70, and 80 patients with the study being stopped as soon as efficacy or futility was concluded based on Bayesian criteria. The probability of falsely concluding efficacy in the absence of an effect was 2.5% yielding desired control of type I error. The empirical power was 79% when true effect corresponded to a Cohen’s *d* of 0.7. To account for a 20% drop-out rate, the maximum sample size was set at 100. Further details of the Bayesian approach and stopping criteria are provided in the Statistical analysis section and Supplementary Materials.

### Statistical Analysis

The primary analysis for efficacy was based on the CIS-fatigue score at 2 weeks after intervention. CIS-fatigue scores are assumed to be normally distributed and a weakly informative conjugate normal-gamma priors was used. Efficacy of bCBT over WLC would be concluded when the posterior probability of the mean CIS-fatigue scores after intervention in the bCBT arm being lower than in the WLC arm exceeded 99%. Futility would be concluded when the predictive probability of concluding efficacy at the maximum sample size of 40 patients per arm dropped below 10%. The posterior mean difference with its 95% credible interval is reported. In addition, Cohen’s *d* is provided as a standardized effect size. Analyses were performed on the intention-to-treat population. Patients who discontinued the intervention or waitlist were still invited to complete the primary outcome assessment. Further details of the Bayesian analysis and frequentist operating characteristics of the design are described in the Supplementary Materials.

Two sensitivity analyses were performed for the primary outcome to assess the robustness of our results: (i) assess the impact of using Bayesian methods as well as restricting the analysis to those with primary endpoint available and (ii) choice of the CIS-fatigue scale as primary endpoint. First, we performed a frequentist analysis of covariance (ANCOVA) analysis in the intention-to-treat population and multiple imputation to account for missing outcomes. Multiple imputation was performed by chained equations (MICE) separately within each treatment group to preserve within-group distributions. The imputation model included the outcome at post-intervention (CIS-fatigue), baseline fatigue (CIS-fatigue), sociodemographic variables (age, sex, educational level), clinical characteristics (tumor hemisphere, WHO grade, AED use, tumor treatment), and baseline depressive symptoms (BDI-pc). Predictive mean matching was used for continuous variables, generating 1000 imputed datasets per treatment group. Imputed datasets were combined for pooled analysis. For a second sensitivity analysis, assessment of the FSS was conducted as an alternative fatigue measure to verify the findings.

Secondary outcomes were compared between groups after treatment and at follow-up using ANCOVA with group allocation as a fixed factor and baseline scores as covariates. Follow-up effects were similarly analyzed using ANCOVA. Different tumor types were not added as covariates as previous work showed no relation between tumor type and fatigue.^53^ Effect sizes were calculated as Cohen’s *d*, interpreted as small (*d < *0.2), medium (*d *= 0.2-0.5), or large (*d *> 0.5).^54^ Two-sided *P-*values <.05 were considered statistically significant. Statistical analyses were performed using RStudio, version 4.3.2.

## Results

### Study Participants

**Figure 1.**
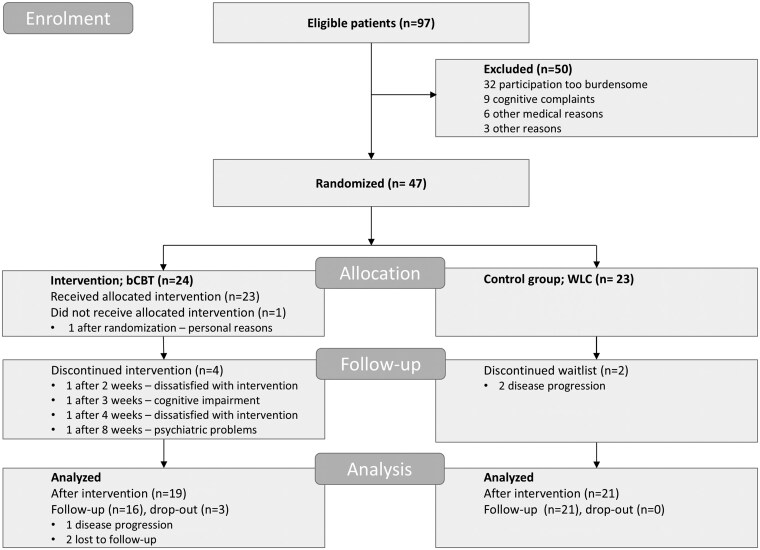
CONSORT flow diagram of RCT. Abbreviations: bCBT, blended cognitive behavioral therapy; CIS, checklist individual strength; WLC, waiting list condition.

Of 97 eligible patients, 47 patients (48%) enrolled and were randomized (Figure 1). Most patients were excluded because participation in a study trial, including hospital visits and study measurements, was too burdensome. Other reasons for exclusion were cognitive complaints and medical conditions that would hinder patients in completing the intervention (ie, not being able to work with a computer or not being able to do graded activity). In the bCBT group, 1 patient withdrew directly after randomization and 4 patients discontinued treatment because they perceived the intervention as unsuitable, or had cognitive or psychiatric problems that hindered participation. In the WLC group, 2 patients withdrew due to tumor progression. In total, 7 patients did not provide primary outcome data (bCBT: *n* = 5; WLC: *n* = 2). Table 1 shows patient characteristics prior to randomization. The average age was 53 years with 47% being female and most patients having had high education (63%).^55^ Most tumors were WHO grade 2 (60%) and were located in the right hemisphere (60%). At the scheduled first interim analysis, 40 patients (19 in the bCBT group and 21 in the WLC group) had completed their assessment at 2 weeks after intervention. Patient characteristics of these 40 patients can be found in Table S1.

**Table 1.** Patient characteristics of all randomized patients

	All (*N* = 47)	Intervention bCBT (*N* = 24)	Waiting list control (*N* = 23)
Age in years, mean (SD)	53 (12)	55 (13)	50 (12)
Sex, no. (%)			
Female	22 (47)	11 (46)	11 (48)
Educational level (Verhage),^a^ no. (%)			
Low (1-4)	6 (13)	3 (12)	3 (13)
Middle (5)	14 (30)	6 (25)	8 (35)
High (6-7)	27 (57)	15 (63)	12 (52)
Tumor location, no. (%)			
Left hemisphere	19 (40)	12 (50)	7 (31)
Right hemisphere	27 (58)	12 (50)	15 (65)
Bilateral	1 (2)	0 (0)	1 (4)
WHO CNS5 classification, no. (%)			
Astrocytoma, IDH-mutant WHO grade 2	13 (28)	6 (25)	7 (30)
Astrocytoma, IDH-mutant WHO grade 3	5 (11)	4 (17)	1 (4)
Astrocytoma, IDH-mutant WHO grade 4	4 (8)	2 (8)	2 (10)
Glioblastoma, IDH-wildtype WHO grade 4	7 (15)	4 (17)	3 (13)
Oligodendroglioma, IDH-mut, 1p/19q-codeleted WHO grade 2	12 (25)	5 (21)	7 (30)
Oligodendroglioma, IDH-mut, 1p/19q-codeleted WHO grade 3	5 (11)	2 (8)	3 (13)
Pleomorphic xanthoastrocytoma/PXA, WHO grade 2, BRAF V600E mutant with CDKN2A/B homozygous deletion	1 (2)	1 (4)	0 (0)
Tumor treatment, no.(%)			
Radiotherapy	36 (77)	18 (75)	18 (78)
Chemotherapy	33 (70)	17 (71)	16 (70)
Surgery			
Resection	42 (89)	21 (88)	21 (90)
Biopsy	5 (11)	3 (12)	2 (10)
Use of AEDs, no. (%)			
Yes	28 (60)	14 (59)	14 (60)
No	4 (8)	2 (8)	2 (10)
Unknown	15 (32)	8 (33)	7 (30)
CIS-fatigue score, mean (SD)	45.94 (6.18)	46.21 (5.67)	45.65 (6.78)
Months since last treatment, mean (SD)	44 (41)	35 (34)	53 (46)

a Education in Verhage educational classification: 1-4 = low (primary/lower vocational), 5 = middle (lower general secondary education), 6-7 = high (higher secondary/university).^55^

Abbreviations: AED, anti-epileptic drugs; CIS-fatigue, fatigue severity subscale score of the Checklist Individual Strength (CIS-20).^46^

### Primary Outcome

**Figure 2.**
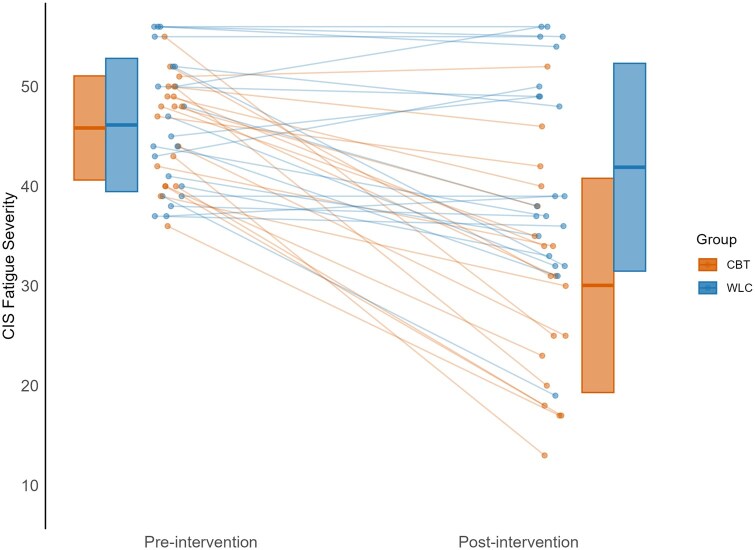
Boxplot of CIS-fatigue severity scores with individual datapoints before and after 12-weeks of bCBT intervention for both groups. Boxes represent mean ± 1 standard deviation (SD) of CIS-fatigue scores. Points show individual participant values; lines connect repeated measurements. Orange colors represent patients in the bCBT group and blue represents patients in the WLC group. CIS-fatigue scores can range between 8 and 56 and a score ≥35 indicates severe fatigue. Abbreviations: CBT, blended cognitive behavioral therapy; WLC, waiting list condition.

After outcomes were available for 40 patients, a first interim analysis took place. Bayesian statistics showed a posterior probability of 0.9994 of mean CIS fatigue scores after treatment being lower in bCBT group compared to WLC group. Therefore, efficacy was concluded and the study was stopped. The posterior mean difference on CIS-fatigue scores after treatment was 11.8 points with 95% credible interval of 5.5-18.5. Cohen’s *d* calculation showed a large estimated effect size (*d *= 1.12) with a 95% confidence interval of 0.43-1.81. Figure 2 shows a boxplot demonstrating the differences in CIS-fatigue severity scores between the 2 groups before and after treatment. The proportion of patients who were no longer severely fatigued after treatment was 68% for bCBT compared to 24% in the WLC group.

A frequentist sensitivity analysis using ANCOVA after multiple imputation yielded an estimate for the mean difference of 11.3 points with 95% confidence interval of 5.6-17.0, *P *< .001, and effect size of *d *= 1.04 (95% CI: 0.39-1.69). Furthermore, using FSS as an alternative measure of fatigue, patients in the bCBT group reported significantly lower FSS fatigue scores than those in the WLC group directly after treatment (*F*(1,37) = 19.80; *P *= .0001, *d *= 1.18).

### Secondary Outcomes

**Figure 3.**
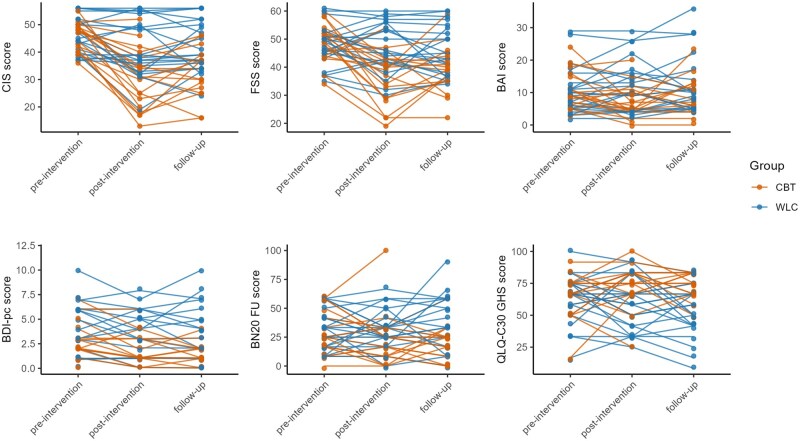
Longitudinal line plots for primary and secondary outcomes. Patients were assessed pre-intervention, post-intervention (2 weeks after intervention) and at follow-up (12 weeks after intervention). Each dot represents a patient. Orange colors represent patients in the bCBT group and blue represents patients in the WLC group. Abbreviations: CBT, blended cognitive behavioral therapy; WLC, waiting list condition.

We found several statistically significant differences in secondary outcomes between the bCBT and WLC group at 2 weeks after treatment and at 12 weeks of follow-up (Figure 3 and Table S2). After treatment and at follow-up, patients in the bCBT group reported better HRQoL than those in the WLC group as indexed by the EORTC-QLQ-C30 Global Health Status scores (after treatment: *F*(1,37) = 5.02; *P *= .031; *d *= −0.68 and follow-up: *F*(1,34) = 13.18; *P *= .0002; *d *= −1.19). As opposed to the WLC group, anxiety scores were significantly lower directly after treatment in the bCBT group (*F*(1,36) = 10.66, *P *= .002; *d *= 0.73), but not at follow-up. Conversely, BN20 Future Uncertainty scores were significantly lower in the bCBT group compared to the WLC group at follow-up (*F*(1,34) = 4.84; *P *= .035; *d *= 0.85), but not directly after treatment. Depressive symptoms assessed with BDI-PC did not reach significance either directly after treatment or at follow-up. Fatigue scores measured with CIS-fatigue severity and the FSS as a sensitivity measure both showed lower fatigue at follow-up in the bCBT group compared to the WLC group (CIS-fatigue: *F*(1,33) = 11.97; *P *= .002; *d *= 1.22, FSS: *F*(1,33) = 11.78; *P *= .0016; *d *= 0.81). The proportion of patients who were no longer severely fatigued at follow-up was 56% for bCBT compared to 24% in the WLC group. All significant differences reflect medium-to-large-sized effects; these effects are displayed in Table 2.

**Table 2.** Effects of bCBT on secondary outcomes

	bCBT vs WLC	
	Post-intervention	Follow-up
	Cohen’s *d* [95% CI]	Cohen’s *d* [95% CI]
Primary outcome		
Fatigue (CIS-fatigue)	1.12 [0.43, 1.81]^a^	1.22 [0.52, 1.92]^a^
Secondary outcomes		
Fatigue (FSS)	1.18 [0.49, 1.88]^a^	0.81 [0.11, 1.51]^a^
HRQoL (QLQ-C30—global health status)	−0.68 [−1.34, −0.019]^a^	−1.19 [−1.91, −0.46]^a^
Anxiety (BAI)	0.73 [0.066, 1.39]^a^	0.42 [−0.26, 1.10]
Depression (BDI—Primary Care)	0.65 [−0.01, 1.31]	0.77 [0.071, 1.47]
Future uncertainty (BN20)	0.54 [−0.12, 1.19]	0.85 [0.15, 1.55]^a^

Effect sizes for primary and secondary outcomes of bCBT versus WLC post-intervention (2 weeks after intervention) and at follow-up (12 weeks after intervention). Effect sizes were calculated as Cohen’s *d*, interpreted as small (*d < *0.2), medium (*d *= 0.2-0.5), or large (*d *> 0.5).^54^

a Represent significant effects (*P *< .05).

Abbreviations: bCBT, blended cognitive behavioral therapy; BAI, Beck Anxiety Index; BDI, Beck Depression Index; BN20, brain tumor-specific HRQOL issues; CIS, checklist individual strength; EORTC-QLQ-30, European Organization for Research and Treatment of Cancer (EORTC) Quality of Life Questionnaire-Core 30; FSS, Fatigue Severity Scale; WLC, waiting list condition.

### Acceptance and Burden of the Intervention

All completers attended the full course of treatment sessions. Patient satisfaction was rated with a mean of 6.6 (SD 2.7) directly after the intervention and 7.3 (SD 1.3) at follow-up (scale 0-10). At these respective time points, 54% and 60% of patients indicated they would recommend the intervention to other glioma patients with severe fatigue, while the remaining patients were uncertain. Qualitative feedback indicated that patients valued the focus on the sleep–wake pattern and the activity list, which many continued to use after the program. Several patients expressed gratitude for reduced fatigue and wished the intervention had lasted longer. Reported challenges included the intensity of the program alongside daily obligations, repetitive or unclear questionnaires, and difficulties maintaining a strict daily rhythm. A minority felt the therapeutic approach did not match their personal coping style, while others suggested clearer instructions and more flexible pacing. Despite these remarks, overall acceptance was high, and the majority considered the intervention useful and supportive. A detailed overview with ratings and qualitative feedback per patients is provided in Table S3.

## Discussion

This Bayesian adaptive RCT evaluated whether a 12-week bCBT intervention reduced severe fatigue in patients with diffuse glioma compared to a waiting list control. The results demonstrate the efficacy of bCBT, as the majority of patients were no longer severely fatigued after the intervention. Additionally, following bCBT, patients reported lower anxiety and future uncertainty, and higher HRQoL, establishing the evidence-basis for this intervention against fatigue in diffuse glioma. Moreover, the positive effects of the intervention were sustained for at least 12 weeks after the intervention, as more than half of the patients following bCBT showed remission of severe fatigue at follow-up. Our findings align with the ASCO guidelines that recommend CBT for post-cancer fatigue and palliative cancer patients,^56^ and extend evidence for CBT from previous trials in primary brain tumors^44^ and patients with malignancies other than brain cancer,^36^^,^^37^ with diseases other than cancer,^38-40^ and with glioma but without severe fatigue.^31^^,^^57^

The present study demonstrates that large effects can be achieved with blended care. This is clinically relevant considering the cognitive, neurological, and logistical barriers that glioma patients encounter to participate in face-to-face treatment. Although not yet studied directly in this population, evidence from other groups indicates that blended CBT offers comparable efficacy to traditional formats, with added flexibility, efficiency, and reduced burden.^58-60^ The effectiveness of bCBT in reducing severe fatigue in patients with glioma, as compared to a WLC, may be explained by its targeted and tailored approach to modifiable perpetuating factors such as unhelpful beliefs, disrupted sleep, and reduced activity levels.^61^^,^^62^ This aligns with findings that fatigue in glioma is more strongly associated with psychological and behavioral factors than with tumor- or treatment-related factors.^53^ These results support the cognitive-behavioral model of fatigue,^62-64^ which posits that while fatigue may be initially triggered by the tumor or its treatment, its persistence is maintained by modifiable cognitive and behavioral processes. The blended format combines the structure and accountability of face-to-face sessions with the flexibility of online components, which may enhance engagement and accessibility, especially for patients with cognitive or physical limitations.^65^ Moreover, CBT promotes self-management and a sense of control over symptoms, which may counteract the helplessness often associated with chronic fatigue in brain tumor patients.^66^ The observed improvements may also reflect broader psychological benefits of CBT, such as reductions in comorbid anxiety, which is known to exacerbate fatigue.^61^ Moderate-to-high satisfaction ratings and the majority willingness to recommend this intervention support its feasibility and acceptability. At the same time, qualitative feedback highlights the need for greater flexibility and possibly longer duration to optimize patient experience and reduce burden.

Beyond patients with diffuse glioma, our findings may inform supportive care for other cancer populations affected by persistent fatigue. Survivors of breast,^36^^,^^42^ colorectal and prostate,^36^ and hematologic cancers—including those who have undergone stem cell transplantation^32^—have shown benefit from CBT-based interventions for fatigue. Additionally, considering the overlap in symptoms, this treatment might also be suitable for patients with CNS involvement, such as meningioma, who often experience fatigue.^67^ The flexibility and individual tailoring of bCBT make it particularly appropriate for these diverse groups, especially those with stable disease and limited access to conventional in-person care.

Limitations of the current study include the inability to blind participants and therapists and the absence of a true placebo condition, which are common issues in psychotherapy research.^68^ The use of a waitlist control may have inflated the observed treatment effect, as participants on a waitlist often show limited improvement or even deterioration, whereas those receiving treatment benefit from both specific and nonspecific therapeutic factors.^68-70^ In the context of our Bayesian multistage design, such inflated contrasts could increase the likelihood of crossing efficacy thresholds at interim analyses, even though overall type I error was strictly controlled. Thus, the effect size estimates should be interpreted cautiously, as they may not generalize to comparisons with active or placebo controls. The current study setup with a waiting list remains a justifiable control condition, considering the severity of fatigue and the lack of effective alternatives for this vulnerable population. Furthermore, the use of a Bayesian trial design allowed for flexible sample sizing and efficacy was concluded at the first interim analysis. However, although efficacy was shown using posterior probabilities, any estimation of treatment effects on primary endpoint after stopping early for efficacy may be susceptible to bias in favor of bCBT.^71^^,^^72^ Sensitivity analyses were conducted for the primary outcome which used all patients randomized and multiple imputation or an alternative fatigue scale. Also, an effect was still seen at 12 weeks follow-up with similar large effect sizes. Importantly, by concluding validated efficacy at the first interim-analysis, fewer vulnerable patients were exposed to a study protocol and other patients were granted accelerated access to effective treatment.

Another limitation concerns several practical challenges encountered during the study. Recruitment proved difficult, as the demanding nature of the study protocol discouraged participation. Moreover, some patients discontinued the study due to disease progression or because the intervention was not well-suited to their individual needs. Two patients were lost to follow-up without clear reason, so their longer-term benefit remains uncertain. In addition, patients with severe cognitive impairments—such as substantial memory, planning, or digital difficulties—were excluded from participation. Lastly, requiring an interval of at least 8 weeks without oncological treatment ensured that patients were in a stable disease phase, but may have reduced the generalizability of our findings, especially for patients with high-grade glioma who often remain on active therapy. In particular, only a small number of patients with IDH-wildtype glioblastoma completed follow-up (*n* = 4), reducing the transferability of our findings to this clinically important subgroup. More broadly, our results apply primarily to a relatively cognitively preserved population in a stable disease phase. Future research should aim for larger GBM samples and examine which patient characteristics predict response to bCBT to support a more personalized and accessible approach to fatigue management for all glioma patients. Notably, 32% of patients who received bCBT were still severely fatigued after treatment. This may reflect variability in underlying fatigue mechanisms, treatment adherence, or patient-specific factors. Understanding predictors of (non)response warrants further investigation to better tailor interventions and improve outcomes. Taken together, these challenges highlight the importance of offering a palette of supportive interventions, of which bCBT for fatigue may serve as a valuable option for selected patients.

This study informs clinical care by demonstrating that blended CBT is effective in reducing severe fatigue in patients with stable diffuse glioma. This is a critical step toward evidence-based care and improved HRQoL for glioma patients. Future steps include testing the intervention in patients in other disease phases and/or with other types of brain tumors.

## Supplementary Material

noaf256_Supplementary_Data

## Data Availability

Data will be made available upon reasonable request.
