# Supplementary material for: Bayesian adaptive randomized trial of blended cognitive behavioral therapy for severe fatigue in stable diffuse glioma
Source: Neuro Oncol. 2025 Nov 9;28(3):693–703. doi: 10.1093/neuonc/noaf256 (PMC13070507; doi:10.1093/neuonc/noaf256)
Supplement: noaf256_Supplementary_Data [file noaf256_supplementary_data.zip › CBT for Fatigue in Diffuse Glioma_supplement_v2_clean.docx]

Supplementary Materials

## Table S1. Patient characteristics of 40 patients who reached the primary endpoint.

|  | | | *All*  *(N=40)* | *Intervention CBT*  *(N=19)* | *Control waitlist*  *(N=21)* |
| --- | --- | --- | --- | --- | --- |
| Age in years, mean (SD) | | | 53 (12) | 55 (12) | 50 (12) |
| Sex, no. (%) | | |  |  |  |
|  | | Female | 20 (50) | 9 (47) | 11 (52) |
| Educational level (Verhage)^1^, no. (%) | | |  |  |  |
|  | Low (1-4) | | 4 (10) | 1 (5) | 3 (14) |
|  | Middle (5) | | 11 (27) | 4 (21) | 7 (33) |
|  | High (6-7) | | 25 (63) | 14 (74) | 11 (53) |
| Tumor location, no. (%) | | |  |  |  |
|  | Left hemisphere | | 15 (37) | 10 (53) | 5 (24) |
|  | Right hemisphere | | 24 (60) | 9 (47) | 15 (71) |
|  | Bilateral | | 1 (3) | 0 (0) | 1 (5) |
| WHO CNS5 classification; no. (%) | | |  |  |  |
|  | | Astrocytoma, IDH-mutant WHO grade 2 | 11 (28) | 5 (26) | 6 (28) |
|  | | Astrocytoma, IDH-mutant WHO grade 3 | 3 (7) | 2 (11) | 1 (5) |
|  | | Astrocytoma, IDH-mutant WHO grade 4 | 3 (7) | 1 (5) | 2 (10) |
|  | | Glioblastoma, IDH-wildtype WHO grade 4 | 5 (12) | 3 (16) | 2 (10) |
|  | | Oligodendroglioma, IDH-mut, 1p/19q-codeleted WHO grade 2 | 12 (30) | 5 (26) | 7 (33) |
|  | | Oligodendroglioma, IDH-mut, 1p/19q-codeleted WHO grade 3 | 5 (13) | 2 (11) | 3 (14) |
|  | | Pleomorphic xanthoastrocytoma /PXA, WHO grade 2, BRAF V600E mutant with CDKN2A/B homozygous deletion | 1 (3) | 1 (5) | 0 (0) |
| Tumor treatment, no.(%) | | |  |  |  |
|  | | Radiotherapy | 32 (80) | 15 (79) | 17 (81) |
|  | | Chemotherapy | 30 (75) | 15 (79) | 15 (71) |
| Surgery | | |  |  |  |
|  | | Resection | 35 (88) | 16 (84) | 19 (90) |
|  | | Biopsy | 5 (12) | 3 (16) | 2 (10) |
| Use of AEDs, no. (%) | | |  |  |  |
|  | Yes | | 22 (55) | 10 (53) | 12 (57) |
|  | No | | 4 (10) | 2 (11) | 2 (10) |
|  | Unknown | | 14 (35) | 7 (36) | 7 (33) |
| CIS-fatigue score, mean (SD) | | | 46 (5.96) | 45.84 | 46.14 |
| Months since last treatment, mean (SD) | | | 48 (43) | 39 (38) | 57 (46) |

^1^Education in Verhage educational classification^1^. AED, Anti-epileptic drugs; CIS-fatigue, fatigue severity subscale score of the Checklist Individual Strength (CIS-20)^2^.

## Table S2. ANCOVA for primary and secondary outcomes.

|  | Pre-intervention | Post-intervention | Follow-up | CBT vs WLC  Post-intervention | CBT vs WLC  Follow-up |
| --- | --- | --- | --- | --- | --- |
|  | *Mean ± SD* | *Mean ± SD* | *Mean ± SD* | *F-metric, p-value; d (effect size)* | *F-metric, p-value; d (effect size)* |
| Primary ouctome | |  |  |  |  |
| CIS – fatigue severity | |  |  |  |  |
|  | CBT: 45.84 ± 5.22  WLC: 46.14 ± 6.68 | **CBT: 30.05 ± 10.74**  WLC: 41.90 ± 10.42 | **CBT: 32.25 ± 9.25**  WLC: 43.10 ± 8.43 | *F*(1,37)=18.49; *p=*0.0001; *d*=1.12 | *F*(1,33)=11.97; *p*=0.002; *d*=1.22 |
| Secondary outcomes | |  |  |  |  |
| FSS | |  |  |  |  |
|  | CBT: 49.11 ± 6.82  WLC: 47.90 ± 7.44 | **CBT: 36.26 ± 9.46**  WLC: 47.29 ± 9.20 | **CBT: 39.06 ± 8.37**  WLC: 46.70 ± 8.71 | *F*(1,37)=19.80; *p*=0.0001; *d*=1.18 | *F*(1,33)=11.78; *p*=0.0016; *d*=1.22 |
| QLQ-C30 – Global Health Status | | |  |  |  |
|  | CBT: 64.91 ± 16.57  WLC: 63.91 ± 19.82 | **CBT: 71.93 ± 16.94**  WLC: 58.73 ± 21.49 | **CBT: 93.74 ± 10.19**  WLC: 51.25 ± 20.64 | *F*(1,37)=5.02; *p*=0.031; *d*=-0.68 | *F*(1,34)=13.18; *p*=0.0002; *d*=-1.19 |
| BAI | |  |  |  |  |
|  | CBT: 10.37 ± 5.64  WLC: 10.95 ± 7.65 | **CBT: 7.16 ± 4.95**  WLC: 12.14 ± 8.19 | CBT: 8.81 ± 5.98  WLC: 12.5 ± 9.13 | *F*(1,36)=10.66, *p*=0.002; *d*=0.73 | *F*(1,33)=3.08; *p*=0.088; *d*=0.42 |
| BDI – Primary care | |  |  |  |  |
|  | CBT: 3.05 ± 2.63  WLC: 3.95 ± 2.67 | CBT: 1.74 ± 1.66  WLC: 3.19 ± 2.64 | CBT: 1.31 ± 1.14  WLC: 3.35 ± 3.07 | *F*(1,37)=3.308; *p*=0.077; *d*=0.65 | *F*(1,33)=3.6; *p*=0.066; *d*=0.77 |
| BN20 – Future Uncertainty | |  |  |  |  |
|  | CBT: 27.19 ± 18.15  WLC: 32.94 ± 17.17 | CBT: 22.37 ± 22.58  WLC: 32.94 ± 16.77 | **CBT: 19.79 ± 15.18**  WLC: 36.90 ± 23.21 | *F*(1,37)=1.735; *p*=0.200; *d=*0.54 | *F*(1,34)=4.84; *p*=0.035; *d*=0.85 |

Secondary outcomes were compared between groups using ANCOVA with group allocation as a fixed factor and baseline scores as covariates at post-intervention (2 weeks after intervention) and at follow-up (12 weeks after intervention). Bold numbers represent significant better outcomes (p < 0.05). ES, effect size estimated with Cohen’s *d* small (d<0.2), medium (d=0.2-0.5), or large (d>0.5)^3^; CBT, blended cognitive behavioral therapy; WLC, waiting list condition; CIS, Checklist Individual Strength; EORTC-QLQ-30, European Organization for Research and Treatment of Cancer (EORTC) Quality of Life Questionnaire-Core 30; BAI, Beck Anxiety Index; BDI, Beck Depression Index; BN20, Brain tumor-specific HRQOL issues.

## Table S3. Qualitative assessment of intervention.

| ID | Stopped intervention | Satisfaction score | | | Time investment score | Recommend treatment | | Advises for improvement |
| --- | --- | --- | --- | --- | --- | --- | --- | --- |
| *After treatment* | | | | | | | | |
| 4 | No | | 6 | 8 | | | Yes | “For me it was not a solution, and by not sleeping during the day I ended up going to bed early in the evening. If I do nap during the day, I have energy in the evening and also cook. Without napping I am so terribly tired that I don’t cook, just eat a few crackers, and want to go to bed by 9:30 p.m. I do sleep less now. Sometimes only an hour, or I lie awake for an hour. That helps.” |
| 7 | No | | 8 | 9 | | | Yes | “I am curious about the follow-up. I would also like to receive some tips about (online) courses for further depth.” |
| 9 | No | | 6 | 6 | | | Yes | “The questionnaires are not always very clear and do not always relate to my problem.” |
| 16 | Yes | | 8 | 10 | | | Yes | “I did not complete the treatment because of a fall, during which my shoulder was dislocated. Unfortunately!!” |
| 20 | No | | 7 | 7 | | | Uncertain | “No.” |
| 25 | No | | 3 | 4 | | | Uncertain | “More open questions” |
| 32 | No | | 10 | 7 | | | Yes | “I wish the treatment had lasted longer. I would have liked to take a break while building up the activities, because I felt emotionally tired. This fatigue arose from suddenly doing too much.” |
| 36 | No | | 6 | 6 | | | Uncertain | “I had difficulty with the questions about thoughts related to fatigue, as these came up every day. I did find the focus on the sleep–wake rhythm useful (it made me more consistent in going to bed on time). The exercise program aimed at building up energy through movement—in my case walking—was very helpful: starting each day with 5 × 1 minute and increasing by 1 minute each day. Unfortunately, when I reached 5 × 15 minutes this came to an abrupt end due to a bad fall, and I was only able to resume walking after three weeks.” |
| 49 | Yes | | 6 | 3 | | | Uncertain | “I have already shared this with my therapist.” |
| 52 | No | | 6 | 5 | | | Uncertain | “There is a lot of personal attention, but … I don’t really know what exactly it has done for me.” |
| 57 | No | | 9 | 8 | | | Yes | “1. A video call every other week felt like the minimum to me. Less would really have been too little—you really need support to implement this program, otherwise it stagnates and you get stuck. Emailing is not enough. 2. It was mentioned beforehand that the program would be intensive, but not to what extent. That could have been clearer. At times I found it quite a lot on top of daily life and obligations, which of course also continue. 3. For me the program could have been longer, around 4–6 months. The severe fatigue is gone (hooray!), but the chapter on work resumption, for example, is really not yet finished. I would have liked guidance with that. It is a pity that the program ends now and that there are no other lines of support after this study. 4. Please also provide the course material in a PDF so that it can be easily printed; now it was often difficult to find where something was again. Apart from that, only compliments! You have helped me enormously in a short time. Bravo!” |
| 60 | No | | 10 | 10 | | | Yes | “No, not really. Because my tips do not necessarily help someone else. It’s personal.” |
| 62 | Yes | | 1 | 1 | | | Uncertain | “I stopped right away because it wasn’t for me. I found it too ‘floaty’/‘woolly’.” |
| *At follow up* | | | | | | | | |
| 4 | No | | 8 | 8 | | | Yes | “For some people it helps better than for others.” |
| 7 | No | | 8 | 10 | | | Yes | “I only realized later that there was a supportive online program with various brain exercises presented as games, etc. At first I ignored it, but when I received an email saying that my access would be discontinued, I understood it was meant to support this program. Clearer explanation would have been desirable.” |
| 9 | No | | 7 | 8 | | | Yes | “In the questions it is not always clear whether they refer to physical or mental fatigue.” |
| 16 | Yes | | 7 | 4 | | | Uncertain | “I interrupted the fatigue study midway because of a fall on my left shoulder. Due to the fatigue from that, I could no longer focus on the fatigue study. Regarding the questionnaires etc., the video calls take a lot of time and some of the questions seem similar, which feels as if they are being asked twice.” |
| 20 | No | | 6 | 4 | | | Uncertain | “No.” |
| 25 | No | | 8 | 6 | | | Yes | “More room to indicate changes in my situation along the way.” |
| 32 | No | | 9 | 7 | | | Yes | “The treatment has certainly improved my life a lot. I would have liked to have more time to take a break between modules, because during the activity build-up I became emotionally overloaded. I think a break of about two weeks would have helped me to get more benefit from the last module.” |
| 36 | No | | 5 | 5 | | | Uncertain | “Limit the number of questions (content-wise) — the questions are often repeated. I found the activity list very useful; I still use it a lot.” |
| 48 | No | | 9 | 9 | | | Yes | “Don’t place the part about anxiety at the end.” |
| 62 | Yes | | 6 | 6 | | | Uncertain | “Make clear in advance what it entails. I only heard at my second visit to the UMC that one of the requirements was to keep the same daily rhythm, i.e., going to bed and getting up at the same time every day. I still work four days a week, which means getting up at 05:45, but then I would also have to do that on the other days. That was the main reason I stopped.” |

## Table S4. Intervention content: online modules and assessments.

| Table 2. Content of the online modules and assessments | |
| --- | --- |
| **Online modules** | **Assessments** |
| *1. Introduction and goals*  The patient formulates positive and tangible goals, which consist of activities they want to do when no longer severely fatigued. |  |
| *2. Sleep and rest*  The patient makes a sleep schedule and keeps an online diary with sleep and wake times. A regular sleep–wake cycle and sleep hygiene are discussed. Instructions are given on how to improve these. | - Sickness Impact Profile (subscale sleep and rest) ^4^  - Registration of bedtime, wake-up time and sleep during the day for seven consecutive days |
| 3. *Fatigue related cognitions*  Loss of control over fatigue symptoms and thoughts, fatigue catastrophizing and dysfunctional thoughts are assessed. The patient does exercises to address and change their dysfunctional thoughts and keeps an online diary about these thoughts. Patients learn to focus less on fatigue. | - Fatigue Catastrophizing Scale ^5^  - Illness Management Questionnaire factor III ^6^  - Self Efficacy Scale Fatigue ^7^ |
| *4. Activity regulation*  The patient with a ‘relatively active’ activity pattern learns to distributes activities more evenly. Then both ‘relatively active’ and ‘low active’ patients systematically increase their physical activity with a graded activity program with walking or cycling. They track their daily progress in an online diary. They learn how to solve problems with activity regulation. The module aims to change activity-impeding beliefs and increase the physical activity level of patients. | - With actigraphy (actometer around the ankle for 14 consecutive days) the level of activity is objectified ^8^. The activity pattern will be rated as ‘Low active’ or ‘Relatively active’. |
| *Submodule 4A: Regulation of social activities (optional)* The relationship between cancer, fatigue and a reduction of social activities as well as cognitions about social activities are assessed. The patient increases his/her social activity level. | - Sickness Impact Profile (*indication for this optional submodule: score subscale social activities ≥ 100*) ^4^ |
| *Submodule 4B: Regulation of mental activities (optional)*  The patient learns about cognitive deficits and how to deal with them. The patient increases their mental activity level. | - Checklist Individual Strength *(indication for this optional submodule: score subscale concentration ≥ 18)* ^2^ |
| *Submodule 4C: Going back to work (optional)*  The patient makes a plan to return to work or increase working hours. | *(indication for this optional submodule: if the patient has set a goal to return to work or increase working hours)* |
| *5. Fear of disease progression (optional)*  Thoughts and situations that trigger fear about the future or tumour growth are assessed. The patient learns to be more accepting towards anxious feelings and to handle these feelings with exercises based on detached mindfulness, meta-cognitive therapy and exposure. | - Fear of Progression Questionnaire *(indication for the module: score ≥ 34)* ^9^  - Beck Anxiety Inventory *(indication for the module: score ≥ 36*) ^10^ |
| *6. Social support (optional)*  Reactions of the partner and significant others to fatigue are assessed. Perceived discrepancy between actual and desired social support, experiences with negative social interactions and unrealistic expectations of others are assessed. The goal of this module is to support emotional independence of others and to become more assertive, as far as fatigue is concerned. | - Social Support List, subscale discrepancy *(indication for the module: score ≥ 50*) and subscale negative interactions (*indication for the module: score ≥ 14*) ^11^ |
| *7. Living with a brain tumour (optional)*  This module focuses on uncertainty about the future and how one can deal with the fact that one has an incurable disease. Several elements from meaning-centred psychotherapy, well-being therapy and writing therapy are used to help the patients to deal with the disease trajectory. | - Illness Cognition Questionnaire, subscale acceptance (*indication for the module: score* ≤ 12) and subscale helplessness (*indication for the module: score ≥ 14*) ^12^  - Impact Event Scale, subscale avoidance(*indication for the module: score ≥ 10*) and subscale intrusion(*indication for the module: score ≥ 10*) ^13^ |
| *8. Realizing goals*  The patient looks back at the goals set in the first module and makes a plan to realize these goals. The intervention is evaluated. |  |

##

## Details of the Bayesian analysis.

### Bayesian model and priors

CIS fatigue scores at 14 weeks for patients in the CBT arm are assumed to be independent and identically distributed according to a normal distribution with mean μ_CBT_ and variance σ_CBT_^2^ = 1/τ_CBT_, where the parameters μ_CBT_ and τ_CBT_ are unknown, i.e.

Y^CBT^(j) ~ N(μ_CBT_, σ_CBT_^2^ = 1/τ_CBT_) j = 1, 2,…, 40

A similar assumption is made regarding the outcomes for patients in the usual care arm but with a possibly different mean μ_UC_ and variance σ_UC_^2^ = 1/τ_UC_, i.e.

Y^UC^(j) ~ N(μ_UC,_ σ_UC_^2^ = 1/τ_UC_) j = 1, 2,…, 40

In the Bayesian approach, the unknown means and variances are assumed to be random. For both arms we consider a conjugate normal-gamma prior distribution for the joint distribution of the mean μ and the precision parameter τ. This is a weakly informative prior. For the CBT arm:

μ^CBT^ | τ^CBT^ ~ N(μ_0_^CBT^_,_ 1/(κ_0_τ^CBT^));

τ^CBT^ ~ Gamma (α_0_^CBT^,β_0_^CBT^),

with α_0_^CBT^ and β_0_^CBT^ the shape and inverse scale (hyper)parameters for the prior (gamma) distribution for the precision parameter τ^CBT^ and μ_0_^CBT^ a (hyper)parameter reflecting the prior mean of the fatigue scores. The parameter κ_0_ is used to tune the relative weights given to the prior mean and the mean of the observed data. The same conjugate prior distribution is used for mean and precision the usual care arm, i.e.

μ^UC^| τ^UC^ ~ Normal ( μ_0_^UC^_,_ 1/(κ_0_τ^UC^) );

τ^UC^ ~ Gamma (α_0_^UC^, β_0_^UC^)

### Posterior distributions

As the distribution is conjugate, the posterior is again a gamma-normal distribution. If the prior distribution for (μ, τ) is

μ | τ ~N(μ_0,_ 1/(κ_0_τ));

τ ~ Gamma (α_0_,β_0_)

and observed data observed is given by x_1_, …, x_n_ with average $\bar{x}$ (where the data are assumed to be independent and normally distributed with same mean and variance), then the posterior distribution for (μ, τ) is again a gamma-normal distribution

μ | τ ~N(μ_n,_ 1/(κ_n_τ));

τ ~ Gamma (α_n_,β_n_)

where

μ_n_ = $\frac{\kappa_{0}\mu_{0}+n\bar{x}}{\kappa_{0}+n}$

κ_n_ = κ_0_ + n

α_n_ = α_0_ + n/2

β_n_ = β_0_ + $\frac{1}{2}\sum_{i=1}^{n} {(x_{i}-\bar{x})}^{2}+\frac{\kappa_{0}n{(\bar{x}-\mu_{0})}^{2}}{2\left( \kappa_{0}+n \right)}$

(Murphy, K. Conjugate analysis of the Gaussian distribution. Last updated October 3, 2007, <https://www.cs.ubc.ca/~murphyk/Papers/bayesGauss.pdf> )

### Choice of values for the hyperparameters

We set hyperparameters for the means at μ_0_^CBT^ = μ_0_^UC^= 50 and κ_0_ = 0.001 (given very low weight to prior mean compared to average in the observed data) and set α_0_ ^CBT^= α_0_^UC^ = 0.1 and β_0_^CBT^= β_0_^UC^ = 0.1. Figure 1 shows the posterior distribution for mean and standard deviation for 100 simulated datasets for 20 patients where mean and standard deviation of the data are 39 and 14, respectively. From this figure we see that for this choice of hyperparameter values the influence of the prior on the estimates is negligible.


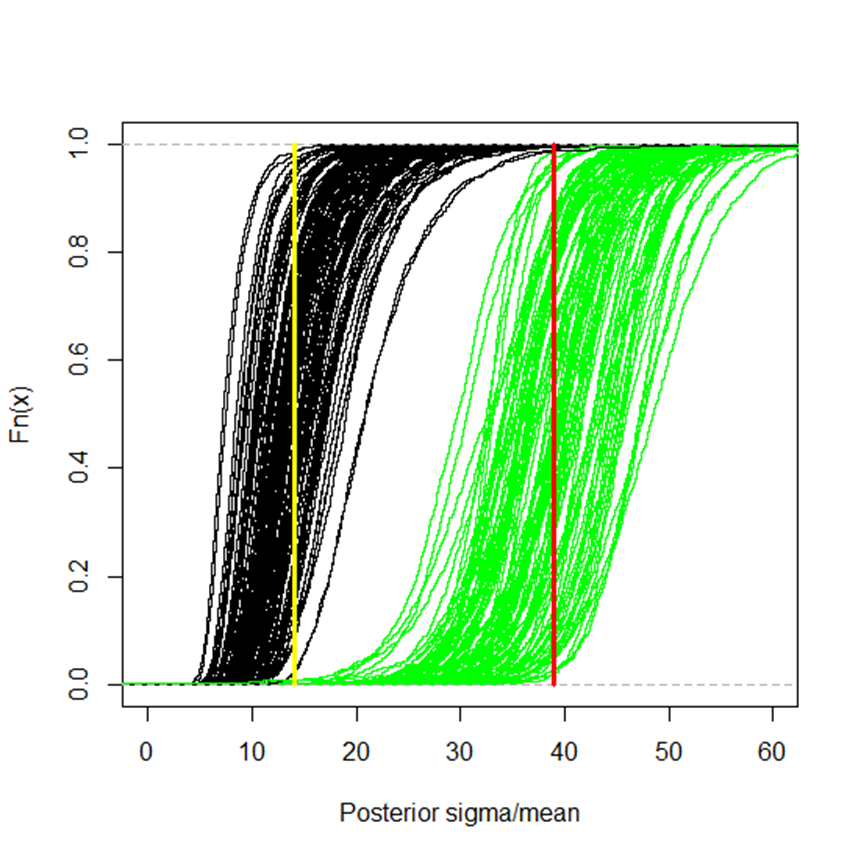


Figure S1: Empirical cumulative posterior density functions for mean (green curves) and standard deviation (black curves) for the weakly informative normal-gamma prior and selected values for hyperparameters for 100 simulated datasets with mean of 39 (red line) and standard deviation of 14 (yellow line).

### Sampling from posterior and predictive distributions

Sampling from the posterior distribution will be done by first drawing τ^CBT^ and subsequently drawing μ^CBT^ conditional on the draw of τ^CBT^. Sampling from predictive distributions will be done by by first drawing τ^CBT^ and subsequently drawing μ^CBT^ conditional on the draw of τ^CBT^ and then drawing an observation Y^CBT^ ~ N(μ_CBT_, σ_CBT_^2^ = 1/τ_CBT_). This is done independently for the bCBT and UC arm.

### Criteria for declaring efficacy and futility

Evaluations for efficacy take place after outcomes have been observed for 40, 50, 60, 70 and 80 patients. At each point we will calculate the posterior probability of the mean CIS fatigue scores at 14 weeks being smaller in the bCBT arm than in the usual care arm. As the distribution is conjugate the posterior distribution is again a gamma-normal distribution. When calculating the posterior probability we first sample τ. If the estimate exceeds 0.99, we will conclude efficacy of the bCBT arm.

Evaluations for futility take place after outcomes have been observed for 40, 50, 60 and 70 patients. At each point we evaluate the posterior predictive distribution of the trial concluding efficacy when outcomes of 80 patients are observed. The trial is stopped for futility when the posterior predictive distribution is below 0.10.

### Frequentist operating characteristics of the Bayesian design

Frequentist operating characteristics of the trial with the interim evaluation and final evaluations as specified were evaluated in a simulation study. We assumed the mean CIS fatigue score at 14 weeks to be 39 in the UC arm and the standard deviation in both arms to be 14 points. We set Cohen’s D respectively at 0, 0.1, 0.2, …, 1.0. We simulated 1000 trials per effect size. Empirical rejections rates (type I error in case Cohen’s D is 0 and power otherwise) and expected sample size per arm were calculated for each effect size (Figure 2).


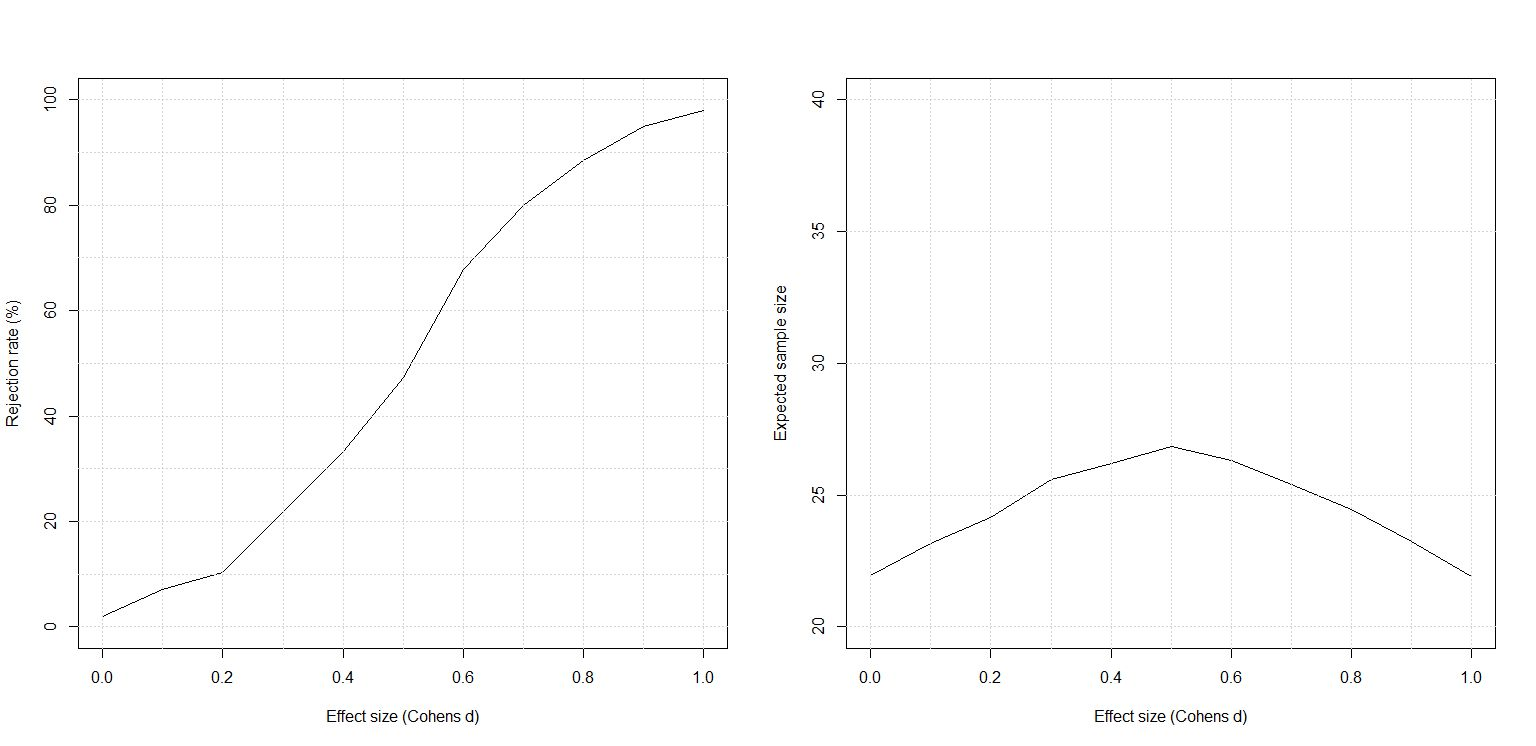


Figure S2: Empirical rejection rate and expected sample size per arm as a function of true effect size

From Figure 2 we see that the trial has the desired power of approximately 80% when Cohen’s D is 0.7. Also, one-sided type I error was found te be controlled at the desired level (empirical type I error was 2.4%). The maximum average sample size was found to be around 27 per arm (54 in total) which was lower than the 34 per arm (68 in total) required for a standard two-arm randomized trials without interim evaluations with the same power.

Empirical type I error and power at Cohen’s D of 0.7 were calculated with higher precision in an independent larger simulation where 25,000 trials were simulated for each of the two effect sizes. The empirical type I error was found to be 2.4% and empirical power was 79% when Cohen’s D is 0.7.

# Supplementary references

1. Verhage F: Intelligentie en leeftijd: Onderzoek bij Nederlanders van twaalf tot zevenenzeventig jaar, van Gorcum Assen, 1964

2. Worm-Smeitink M, Gielissen M, Bloot L, et al: The assessment of fatigue: Psychometric qualities and norms for the Checklist individual strength. J Psychosom Res 98:40-46, 2017

3. Cohen J: Statistical power analysis for the behavioral sciences, routledge, 2013

4. Jacobs HM, Luttik A, Touw-Otten FW, et al: The sickness impact profile; results of an evaluation study of the Dutch version. Ned Tijdschr Geneeskd 134:1950-4, 1990

5. Jacobsen PB, Andrykowski MA, Thors CL: Relationship of catastrophizing to fatigue among women receiving treatment for breast cancer. J Consult Clin Psychol 72:355-61, 2004

6. Ray C, Weir W, Stewart D, et al: Ways of coping with chronic fatigue syndrome: development of an illness management questionnaire. Soc Sci Med 37:385-91, 1993

7. Prins JB, Bleijenberg G, Bazelmans E, et al: Cognitive behaviour therapy for chronic fatigue syndrome: a multicentre randomised controlled trial. The Lancet 357:841-847, 2001

8. Gresham G, Schrack J, Gresham LM, et al: Wearable activity monitors in oncology trials: Current use of an emerging technology. Contemporary Clinical Trials 64:13-21, 2018

9. Goebel S, Mehdorn HM: Fear of disease progression in adult ambulatory patients with brain cancer: prevalence and clinical correlates. Support Care Cancer 27:3521-3529, 2019

10. Ke Y, Ng T, Yeo HL, et al: Psychometric properties and measurement equivalence of the English and Chinese versions of the Beck Anxiety Inventory in patients with breast cancer. Support Care Cancer 25:633-643, 2017

11. Van Sonderen E: Sociale Steun Lijst-Interacties (SSL-I) en Sociale Steun Lijst - Discrepanties (SSL-D). Groningen, Noordelijk Centrum voor Gezondheidsvraagstukken, 1993

12. Evers AW, Kraaimaat FW, Van Lankveld W, et al: Beyond unfavorable thinking: the illness cognition questionnaire for chronic diseases. J Consult Clin Psychol 69:1026-36, 2001

13. Van der Ploeg E, Mooren TT, Kleber RJ, et al: Construct validation of the Dutch version of the impact of event scale. Psychol Assess 16:16-26, 2004
